# The thymic microenvironment gradually modulates the phenotype of thymus‐homing peripheral conventional dendritic cells

**DOI:** 10.1002/iid3.559

**Published:** 2021-11-08

**Authors:** Susanne Herppich, Michael Beckstette, Jochen Huehn

**Affiliations:** ^1^ Department Experimental Immunology Helmholtz Centre for Infection Research Braunschweig Germany; ^2^ Department of Computational Biology for Individualised Medicine Centre for Individualised Infection Medicine, Helmholtz Centre for Infection Research and Hannover Medical School Hannover Germany; ^3^ Present address: Department of Genome Informatics, Faculty of Technology University Bielefeld Bielefeld Germany

**Keywords:** RTOC, splenic cDCs, thymic cDCs, thymus, Treg cell

## Abstract

**Background & Aims:**

Thymic conventional dendritic cells (t‑DCs) are crucial for the development of T cells. A substantial fraction of t‑DCs originates extrathymically and migrates to the thymus. Here, these cells contribute to key processes of central tolerance like the clonal deletion of self‑reactive thymocytes and the generation of regulatory T (Treg) cells. So far, it is only incompletely understood which impact the thymic microenvironment has on thymus‑homing conventional DCs (cDCs), which phenotypic changes occur after the entry of peripheral cDCs into the thymus and which functional properties these modulated cells acquire.

**Materials & Methods:**

In the present study, we mimicked the thymus‑homing of peripheral cDCs by introducing ex vivo isolated splenic cDCs (sp‑DCs) into reaggregated thymic organ cultures (RTOCs).

**Results:**

Already after two days of culture, the transcriptomic profile of sp‑DCs was modulated and had acquired certain key signatures of t‑DCs. The regulated genes included immunomodulatory cytokines and chemokines as well as costimulatory molecules. After four days of culture, sp‑DCs appeared to have at least partially acquired the peculiar Treg cell‐inducing capacity characteristic of t‑DCs.

**Discussion & Conclusion:**

Taken together, our findings indicate that peripheral cDCs possess a high degree of plasticity enabling them to quickly adapt to the thymus‐specific microenvironment. We further provide indirect evidence that thymus‐specific properties such as the efficient induction of Treg cells under homeostatic conditions can be partially transferred to thymus‑homing peripheral cDC subsets.

## Graphical abstract

Thymic conventional dendritic cells (t‐DCs) are crucial for the development of T cells and central tolerance. A substantial fraction of t‐DCs originates extrathymically and migrates to the thymus. Here, we used reaggregated thymic organ cultures (RTOCs) to demonstrate that these thymus‐homing DCs are plastic and can acquire essential thymus‐specific properties upon entry into the thymus.

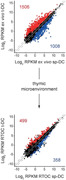

## 1 INTRODUCTION

The thymic microenvironment is formed by a coordinated set of cellular components as well as various soluble proteins establishing a complex network of interactions. Importantly, within this multilayered network, each component is closely connected and interdependent.^1^ Among the cellular components are antigen‐presenting cells (APCs), including thymic epithelial cells (TECs) and thymic conventional dendritic cells (t‐DCs). These have an extraordinary impact on the development of T cells and on the establishment of a functional and highly diverse T cell receptor (TCR) repertoire.^2^ Within the cortex and medulla of the thymus, T cell lineage commitment, as well as a cascade of discrete consecutive differentiation steps, occur. These steps, which lead to the generation of T cells bearing a virtually unlimited number of diverse TCRs, facilitate protection against a vast spectrum of pathogens. However, the elimination of potentially autoreactive clones is required to ensure tolerance towards innocuous and self‐antigens.^3^ Thus, self‐reactive thymocytes are either driven to apoptotic cell death by negative selection or differentiate into regulatory T (Treg) cells, which are characterized by the expression of the transcription factor Foxp3 and essential for the maintenance of immune homeostasis and self‐tolerance.^2^, ^3^, ^4^

Conventional DCs (cDCs) within the thymus play a critical role in the establishment of central tolerance. The compartment of t‐DCs is comprised of approximately 70% cDC1s (CD8α^+^SIRPα^−^ t‐DCs) and 30% cDC2s (CD8α^−^SIRPα^+^ t‐DCs).^5^ Thymic cDC1s, also referred to as resident t‐DCs, arise primarily within the thymus from an early yet undefined thymic progenitor. Due to their XCR1 expression, CD8α^+^SIRPα^−^ t‐DCs colocalize with medullary TECs (mTECs), expressing the XCR1 ligand XCL1.^6^, ^7^, ^8^ Thereby, resident CD8α^+^SIRPα^−^ t‐DCs are able to cross‐present tissue‐restricted antigens that are expressed in mTECs.^9^ In contrast, thymic cDC2s (CD8α^−^SIRPα^+^ t‐DCs) are migratory t‐DCs that originate extrathymically and migrate from the periphery to the thymus. Within the thymus, CD8α^−^SIRPα^+^ t‐DCs are enriched at the corticomedullary junction as well as around small vessels.^10^, ^11^, ^12^ These migratory t‐DCs can capture and display blood‐borne antigens, but also present peripheral antigens.^13^

Accumulating evidence suggests that both resident and migratory t‐DCs play a role in the negative selection and also contribute to the thymic Treg cell development in a nonredundant manner.^2^, ^4^, ^14^, ^15^ Yet, while CD8α^−^SIRPα^+^ migratory t‐DCs were shown to harbor a superior capacity to induce Treg cells in vitro, splenic conventional DCs (sp‐DCs) possess only poor Treg cell induction capacity when compared to bulk t‐DCs.^9^, ^13^, ^16^, ^17^, ^18^ This raises the question of whether the thymic microenvironment has the capacity to modulate the phenotype of thymus‐homing peripheral cDCs and instruct them with efficient Treg cell‐inducing properties.

In the present study, we mimicked the thymus‐homing of peripheral cDCs by introducing ex vivo isolated sp‐DCs into reaggregated thymic organ cultures (RTOCs). This technique provides the possibility to investigate in vitro the interplay of the complex thymic microenvironment with any cell population of interest.^19^ We found that the thymic microenvironment has the capacity to rapidly shift the phenotype of sp‐DCs towards a t‐DC phenotype on transcriptome level. Only a short residence within the RTOC likely improved the Treg cell‐inducing capacity of sp‐DCs, suggesting that the thymic microenvironment harbors a dominant capacity to modulate the functional properties of thymus‐homing peripheral cDCs.

## 2 MATERIAL AND METHODS

### 2.1 Mice

C.SJL(B6)‐*Ptprc* ^*a*^ *Ptprc* ^*b*^/BoyJ mice (CD45.1 congenic mice on BALB/c background, CD45.1xBALB/c mice) and C.Foxp3^tm1(CD2/CD52)Shori^ mice (Foxp3^hCD2^ reporter mice on BALB/c background^20^) were bred and maintained at the central animal facility of the Helmholtz Centre for Infection Research (HZI), which provides state‐of‐the‐art laboratory animal care and service. All mice were housed in barriers under specific pathogen‐free conditions in isolated, ventilated cages, and handled by personnel appropriately trained as well as dedicated animal care staff to assure the highest possible hygienic standards and animal welfare in compliance with German and European animal welfare guidelines. According to the German Animal Welfare Act, sacrificing animals solely to remove organs for scientific purposes is notified to the competent authority. This study was carried out in accordance with recommendations defined by the Federation of European Laboratory Animal Science Associations and the German Animal Welfare Body Society for Laboratory Animal Science using approved protocols.

### 2.2 Antibodies and flow cytometry

Cell suspensions were labeled directly with the following fluorochrome‐conjugated anti‐mouse antibodies purchased from either BioLegend, BD Biosciences, or eBioscience: CD3ε (500A2), CD4 (RM4‐5), CD8α (53‐6.7), CD11c (N418), CD19 (6D5), CD25 (PC61.5), CD44 (IM7), CD45 (30‐F11), CD45.1 (A20), CD45.2 (104), CD49b (DX5), CD80 (16‐10A1), CD90.2 (53‐2.1), CD172α (P84), CD326 (G8.8), anti‐human CD2 (RPA‐2.10), Ly51 (6C3), F4/80 (BM8), I‐A/I‐E (M5/114.15.2), and XCR1 (ZET). UEA‐1 was labeled with a biotinylated anti‐mouse antibody (clone U1216; Vector Labs) and subsequently detected with a fluorochrome‐conjugated streptavidin. To block Fc receptors, the staining mix always contained unconjugated anti‐FcRγIII/II antibody (BioXcell; final concentration 10 µg ml^−1^). For exclusion of dead cells, either 4′,6‐diamidine‐2′‐phenylindole dihydrochloride (Merck) or LIVE/DEAD™ Fixable Near‐IR Stain Kit (Invitrogen) was used. Stained cells were assessed by LSRFortessa™ flow cytometer (BD Biosciences) and data was analyzed with FlowJo® Software (TreeStar).

### 2.3 Isolation of CD4SP Foxp3^−^ thymocytes

CD4^+^CD8^−^ single‐positive (SP) Foxp3^−^ thymocytes were isolated from 4‐ to 6‐week‐old male Foxp3^hCD2^ reporter mice (BALB/c background). Single‐cell suspensions from thymi were labeled with anti‐hCD2‐FITC‐conjugated antibody, anti‐CD4‐PacificBlue‐conjugated antibody, anti‐CD8α‐APC‐conjugated antibody, and anti‐APC microbeads (Miltenyi Biotec). Using the autoMACS® Pro Separation System (Miltenyi Biotec), APC‐labeled CD8α^+^ cells were depleted. From the negative fraction, CD4SP Foxp3^hCD2^^−^ thymocytes were sorted using a FACSAria™ II (BD Biosciences), a FACSAria™ (BD Biosciences), or a MoFlo XDP (Beckman Coulter).

### 2.4 Isolation of DCs

Sp‐DCs and t‐DCs were isolated from 4‐ to 8‐week‐old CD45.1xBALB/c mice. For low‐input RNA sequencing (RNAseq) experiments, female CD45.1xBALB/c mice were used for sp‐DC and t‐DC isolation. In all other experiments, male CD45.1xBALB/c mice were used for sp‐DC and t‐DC isolation. After removal of connective tissue, organs were disrupted with the help of scalpels. The tissue fragments were digested in prewarmed Roswell Park Memorial Institute medium (RPMI; Life Technologies) completed with 10% fetal calf serum (FCS), 50 U ml^−1^ penicillin, 50 U ml^−1^ streptomycin, 25 mM HEPES, 1 mM sodium pyruvate (all Biochrom AG), and 50 μM β‐mercaptoethanol (Life Technologies) (complete RPMI [cRPMI]), containing 2 mg ml^−1^ collagenase/dispase (Roche) and 0.2 mg ml^−1^ DNase I (Roche) at 37°C for 40 min. Digests were filtered through a nylon mesh with pore size of 100 μm (Greiner Bio‐One International GmbH) and subjected to a density gradient using high‐density Easycoll (1.115 g ml^−1^; Biochrom AG) and low‐density Easycoll (1.06 g ml^−1^; Biochrom AG). The gradient was centrifuged at 1350*g*, 4°C with low acceleration and deceleration for 30 min. The low‐density interface was collected and cells were stained with respective fluorochrome‐conjugated antibodies. Finally, t‐DCs were sorted as CD49b^−^F4/80^−^CD90.2^−^CD45.1^+^CD11c^hi^ cells and sp‐DCs were sorted as CD49b^−^F4/80^−^CD3^−^CD19^−^CD45.1^+^CD11c^hi^ cells using a FACSAria™ II (BD Biosciences) or a FACSAria™ (BD Biosciences).

### 2.5 Reaggregated thymic organ cultures

To prepare RTOCs, thymi were isolated from E14.5 to E16.5 fetuses of Foxp3^hCD2^ reporter mice (BALB/c background), pooled and digested in cRPMI containing 0.75 mg ml^−^^1^ collagenase/dispase (Roche) at 37°C for 35 min. Digestion was stopped by the addition of excess medium, and the cell suspension was filtered through a nylon mesh with a pore size of 100 μm. A total of 0.65 × 10^6^ cells were pelleted by centrifugation in a 1.5 ml Eppendorf tube. After complete removal of the supernatant, the cell pellet was dispersed into a slurry and was finally deposited as a freestanding drop on a membrane using a 0.5–2.5 µl Eppendorf pipette. RTOCs were cultured on Whatman® Nuclepore Track‐Etched Membrane (0.8 µm pore size, 13 mm diameter; Merck) placed on a sterilized foam sponge (5 mm thick) in one well of a six‐well plate (Sarstedt) containing 4 ml Dulbecco's modified Eagle's medium supplemented with 50 µg ml^−1^ penicillin, 50 µg ml^−1^ streptomycin, 50 µM β‐mercaptoethanol, 1 mM nonessential amino acids (all from Life Technologies), 10% FCS, and 10 mM HEPES (both from Biochrom AG). Culture conditions were 37°C and 5% CO_2_.

For phenotypic characterization, RTOCs were collected with forceps and 2–3 pooled RTOCs were prepared for analysis on Day 2 and Day 4. RTOCs analyzed for the composition of the T cell compartment were mechanically disrupted with the help of a plunger and a nylon mesh with a pore size of 100 μm. For the analysis of the composition of the TEC compartment, RTOCs were digested in cRPMI (Life Technologies) supplemented with 1 mg ml^−1^ collagenase/dispase (Roche) and 0.1 mg ml^−1^ DNase I (Roche) with gentle rocking at 37°C for 30 min. To support disaggregation of the RTOCs, the digestion suspension was mixed by pipetting every 5–10 min during incubation.

RTOCs containing cDCs were prepared by addition of 0.1 × 10^6^ sorted sp‐DCs or t‐DCs to the 0.65 × 10^6^ cells from the fetal thymi before pelleting. To perform low‐input RNAseq on the inserted sp‐DCs and t‐DCs, RTOCs were harvested on Day 2, 2–3 RTOCs were pooled and processed by digestion.

For the set‐up of syngenic RTOC cocultures, 0.65 × 10^6^ cells from the fetal thymi were mixed with 0.5 × 10^5^ Cell Trace Violet (CTV)™ (Invitrogen)‐labeled sorted CD4SP Foxp3^hCD2^^−^ thymocytes and 0.1 × 10^6^ sorted sp‐DCs or t‐DCs before pelleting. On Day 4, RTOCs were collected with forceps, 2–3 RTOCs were pooled and processed by mechanical disruption for flow cytometric analysis.

### 2.6 Low‐input RNAseq

Total RNA from 1–2 × 10^3^ CD45.1^+^Lin^−^CD11c^hi^ cDCs, which were either isolated directly ex vivo or reisolated from RTOCs, was obtained using the RNeasy® Plus Micro Kit (Qiagen). Complementary DNA (cDNA) was synthesized from 1 ng RNA using the SMART‐Seq® v4 Ultra® Low Input RNA Kit for Sequencing (Takara Bio Europe SAS). Libraries were prepared from the purified cDNA using the Nextera™ XT DNA Library Preparation Kit (Illumina). As input, 0.1 ng cDNA was used per sample and the libraries were cleaned up with 1.8X AMPure® XP beads (Beckman Coulter). Quality and integrity of nucleic acids were assessed using the Agilent Technologies 2100 Bioanalyzer (Agilent Technologies) after each step. The generated libraries were sequenced at the Genome Analytics facility of the HZI on an Illumina HiSeq2500 using 50 bp single reads. The sequenced libraries were assessed for reading quality with the FastQC tool (http://www.bioinformatics.babraham.ac.uk/projects/fastqc), and alignment of the libraries versus the mouse reference genome (Assembly: GRCm38.p6) was performed using the splice junction mapper TopHat2 v1.2.0 ^21^ with default parameterization. Subsequently, DESeq2^22^ was used to compute the differential gene expression between the four different conditions from the read counts. The differentially expressed genes (DEGs) were filtered with a conservative absolute log_2_ fold change cutoff of at least 1.0 and a *p*‐value cutoff, corrected for multiple testing, of at most 0.05. In addition, based on the variance among samples, the reads per kilobase of transcript length per million mapped reads values were calculated.

### 2.7 Statistical analysis

The GraphPad Prism software v7.0 (Graph‐Pad) was used to perform all statistical analyses. Data are presented as mean ± standard deviation For comparison of unmatched groups, a two‐tailed Mann–Whitney test was applied and the *p*‐values were calculated with a long‐rank test (Mantel–Cox). If comparing more than three groups, Kruskal–Wallis test was used. A *p *< .05 was considered as significant; *p < .05; **p < .01; ***p < .001; ****p < .0001.

## 3 RESULTS

### 3.1 RTOCs mature over time and successfully support thymocyte and TEC development

**Figure 1:**
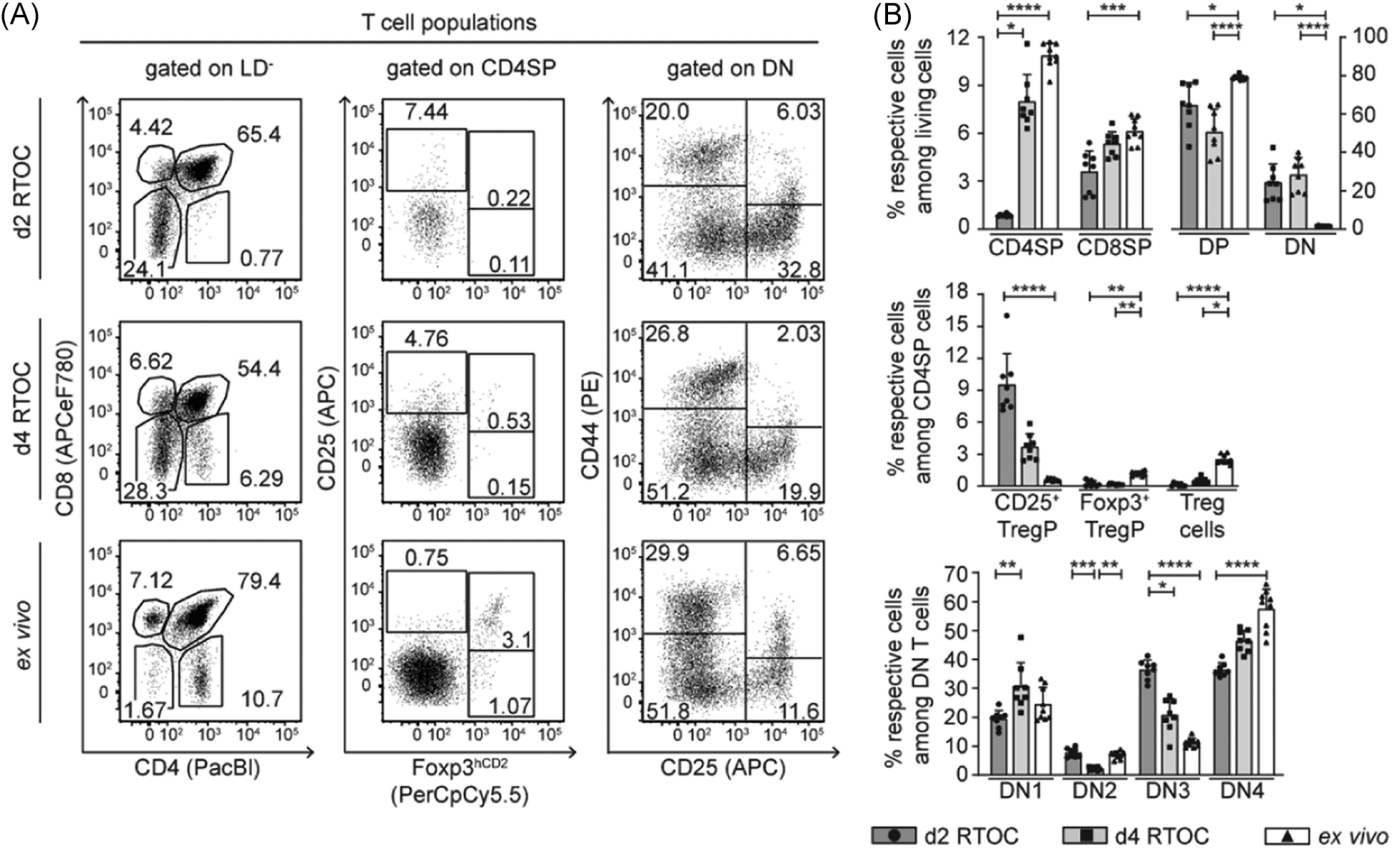
The thymocyte compartment within RTOCs develops over time, approaching population frequencies of adult thymi. RTOCs were generated from single‐cell suspensions of pooled thymi isolated from E14.5 to E16.5 fetuses of Foxp3^hCD2^ reporter mice (BALB/c background). After 2 (d2 RTOCs) or 4 days (d4 RTOCs), RTOCs were harvested and the thymocyte composition was analyzed by flow cytometry. Ex vivo isolated thymi from 4‐ to 6‐week‐old male Foxp3^hCD2^ reporter mice were analyzed as controls. (A) Representative dot plots of thymocyte populations. Numbers indicate the frequencies of cells within the depicted gates. Cells were gated on live/dead (LD)‐negative cells. (B) Graphs show frequencies of indicated thymocyte populations. Data are summarized from two to three independent experiments (mean ± SD; *n* = 3–5 biological replicates per experiment per group; *n* = 6–9 biological replicates per group in total). Data of each cell population was independently tested for significance using Kruskal–Wallis test. Significant differences were indicated by *p < .05; **p < .01; ***p < .001; ****p < .0001. DN, double negative; DP, double positive; RTOC, reaggregated thymic organ culture; Treg cell, regulatory T cell

To use RTOCs as a tool to study the impact of the thymic microenvironment on thymus‐homing peripheral cDCs, we first had to ensure that RTOCs roughly mimic the overall cellular composition of a thymus under steady‐state conditions. For this purpose, we examined by flow cytometry (see Figure S1) the frequencies of major thymocyte and TEC populations within RTOCs harvested on Day 2 and Day 4 of culture, and compared them to ex vivo isolated adult thymi. Thymocytes constitute the largest cell population within a thymus. They develop from thymus‐seeding progenitors via different CD4^−^CD8^−^ double‐negative (DN) stages into CD4^+^CD8^+^ double‐positive (DP) thymocytes, which then further differentiate into either CD4SP or CD8SP cells.^23^ While RTOCs harvested on Day 2 possessed only a low proportion of CD4SP and CD8SP thymocytes, which differs significantly from the thymocyte subset composition in ex vivo isolated adult thymi, frequencies of SP thymocytes increased during 2 further days of culture, reaching levels similar to those found in adult thymi (Figure 1A,B). Within the CD4SP thymocyte population, the frequency of CD25^+^Foxp3^hCD2+^ Treg cells was significantly reduced in RTOCs when compared to ex vivo isolated adult thymi, yet also showing a slight increase over time (Figure 1A,B). Similarly, the frequency of CD25^−^Foxp3^hCD2+^ Treg cell precursors (Foxp3^+^ TregP) also differs significantly between RTOCs harvested on both Day 2 and Day 4 when compared to ex vivo isolated adult thymi. In contrast, the frequency of CD25^+^Foxp3^hCD2^^−^ Treg cell precursors (CD25^+^ TregP) was strongly decreasing over time with significant differences between RTOCs and ex vivo isolated adult thymi observable at Day 2 of the culture. Thus, the process of Treg cell maturation within RTOCs seemed to be slower when compared to the maturation of conventional T cells (Figure 1A,B). Although the population of DP and DN thymocytes did not follow this overall scheme of maturation, the late DN subpopulations (DN3 and DN4) on Day 4 of the culture, separated by the divergent expression of CD25 and CD44, again were closer to frequencies found in adult thymi (Figure 1A,B). Viewed as a whole, RTOCs support the development and maturation of major thymocyte populations. Yet, mild alterations between RTOCs harvested at Day 4 and adult thymi can still be observed for thymocytes, especially for CD4SP and DN thymocyte subsets.

**Figure 2:**
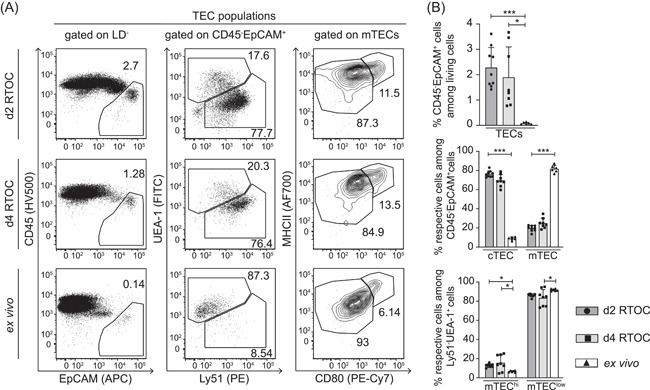
The TEC compartment reaches frequencies found in adult thymi more slowly. RTOCs were generated from single‐cell suspensions of pooled thymi isolated from E14.5 to E16.5 fetuses of Foxp3^hCD2^ reporter mice (BALB/c background). After 2 (d2 RTOCs) or 4 days (d4 RTOCs), RTOCs were harvested and the TEC composition was analyzed by flow cytometry. Ex vivo isolated thymi from 4‐ to 6‐week‐old male Foxp3^hCD2^ reporter mice were analyzed as controls. (A) Representative dot plots for TEC populations. Numbers indicate the frequencies of cells within the depicted gates. Cells were gated on live/dead (LD)‐negative cells. (B) Graphs show the frequency of indicated TEC populations. Data are summarized from two to three independent experiments (mean ± SD; *n* = 3–5 biological replicates per experiment per group; *n* = 6–9 biological replicates per group in total). Data of each cell population was independently tested for significance using the Kruskal–Wallis test. Significant differences were indicated by *p < .05; **p < .01; ***p < .001. cTEC, cortical TEC; MHCII, major histocompatibility complex class II; mTEC, medullary TEC; RTOC, reaggregated thymic organ culture; TEC, thymic epithelial cell

TECs are another important cellular component of the thymus. These CD45^−^EpCAM^+^ cells constitute the scaffold of the thymus and strongly interact with thymocytes and t‐DCs. It is well‐known that the fraction of TECs among total thymic cells decreases early during ontogeny, while the amount of developing thymocytes increases.^1^, ^24^ This general phenomenon was also observed in the present study as the frequency of CD45^−^EpCAM^+^ TECs among total thymic cells was higher in RTOCs harvested on both Day 2 and Day 4 when compared to adult thymi (Figure 2A,B). TECs consist of two main subsets, cortical TECs (cTECs) and mTECs, named by their localization within the two heterogeneous morphological regions of the thymus—cortex and medulla.^25^ cTECs and mTECs were identified as Ly51^+^UEA‐1^−^ and Ly51^−^UEA‐1^+^, respectively, among CD45^−^EpCAM^+^ cells.^26^ RTOCs harvested on Day 2 and Day 4 both showed a high frequency of cTECs and a lower frequency of mTECs among total TECs, while the opposite was observed in ex vivo isolated adult thymi (Figure 2A,B). Although cTECs tended to decline and mTECs increase within RTOCs over time, it becomes obvious that these cell populations reached frequencies found in adult thymi noticeably slower when compared to the abovementioned thymocyte populations. Thus, RTOCs harvested on Day 4 still showed a reversed ratio of the two TEC populations when compared to ex vivo isolated adult thymi. However, both RTOCs harvested on Day 2 and Day 4 already showed similar frequencies of mTEC subpopulations, as defined by the differential expression of CD80 and MHC II,^27^ when compared to ex vivo isolated adult thymi (Figure 2A,B). Overall, RTOCs mature over time and possess the capacity to successfully support thymocyte and TEC development. Thus, they are a versatile tool to study diverse aspects of the thymic microenvironment in vitro. Yet, it has to be considered that the complete establishment of the TEC compartment takes longer and cannot be completed during the short culture period of the RTOCs.

### 3.2 Introducing sp‐DCs into RTOCs does not promote Treg cell differentiation

**Figure 3:**
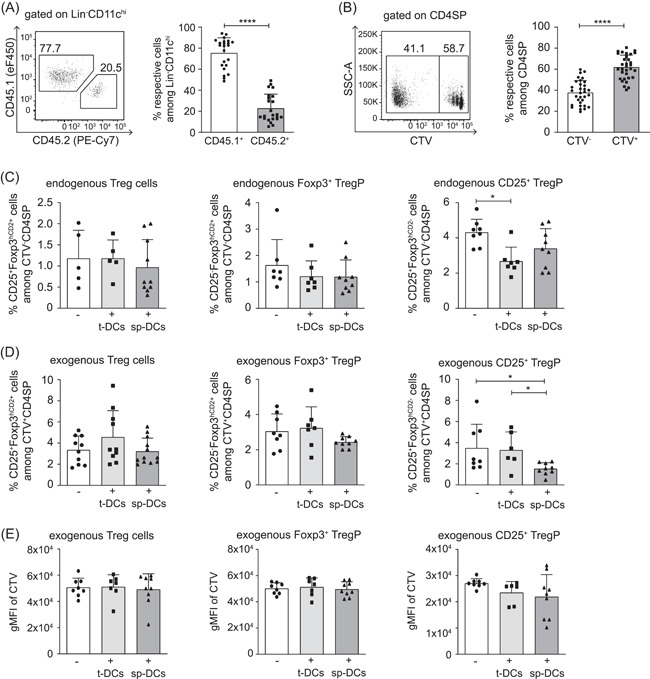
Treg cell frequencies in RTOCs are not affected by introduced sp‐DCs. RTOCs were generated from single‐cell suspensions of pooled thymi isolated from E14.5 to E16.5 fetuses of Foxp3^hCD2^ reporter mice (BALB/c background), and CTV‐labeled CD4SP Foxp3^−^ thymocytes sorted from 4‐ to 6‐week‐old male Foxp3^hCD2^ reporter mice (BALB/c background) as well as sp‐DCs or t‐DCs sorted from 4‐ to 8‐week‐old male CD45.1xBALB/c mice were introduced. (A) Introduced cDCs were identified by the congenic marker CD45.1 in the total cDC pool within an RTOC. Representative dot plot (left) and summarizing graph (right) depict frequencies of exogenous (CD45.1^+^) and endogenous (CD45.2^+^) cDCs among Lin^−^CD11c^hi^ cells, respectively. For the representative dot plot, numbers indicate the frequencies of cells within the depicted gates. For the summarizing graph, data are summarized from two independent experiments (mean ± SD; *n* = 8–15 biological replicates per experiment per group; *n* = 23 biological replicates per group in total) and tested for significance using Mann–Whitney test; ****p < .0001. (B) Exogenously added CD4SP thymocytes can be accurately distinguished from endogenous CD4SP thymocytes with the help of the CTV label. Representative dot plot (left) and summarizing graph (right) depict the frequency of CTV^+^ and CTV^−^ cells among CD4SP thymocytes within RTOCs analyzed by flow cytometry on Day 4. For the representative dot plot, numbers indicate the frequencies of cells within the depicted gates. For the summarizing graph, data were pooled from RTOCs that contain only CTV‐labeled CD4SP thymocytes and RTOCs additionally containing congenically marked t‐DCs or sp‐DCs from four independent experiments (mean ± SD; *n* = 7–9 biological replicates per experiment per group; *n* = 32 biological replicates per group in total) and tested for significance using Mann–Whitney test; ****p < .0001. (C–E) CTV‐labeled CD4SP thymocytes alone or together with sp‐DCs and t‐DCs, respectively, were introduced into an RTOC. On Day 4 of culture, the frequency of CD25^+^Foxp3^hCD2+^ Treg cells, CD25^−^Foxp3^hCD2+^ Treg cell precursors (Foxp3^+^ TregP), and CD25^+^Foxp3^hCD2^^−^ Treg cell precursors (CD25^+^ TregP) among CTV^−^ (RTOC endogenous) (C) and CTV^+^ (RTOC exogenous) (D) CD4SP thymocytes were analyzed by flow cytometry. (E) On Day 4 after set up, the gMFI of CTV among CD25^+^Foxp3^hCD2+^ Treg cells, CD25^−^Foxp3^hCD2+^ Treg cell precursors (Foxp3^+^ TregP) and CD25^+^Foxp3^hCD2‐^ Treg cell precursors (CD25^+^ TregP) among exogenously added CD4SP thymocytes was analyzed by flow cytometry. For (C–E), data are summarized from four independent experiments (mean ± SD; *n* = 1–3 biological replicates per experiment per group; *n* = 5–12 biological replicates per group in total). Data were tested for significance using the Kruskal–Wallis test. Significant differences were indicated by *p < .05. cDC, conventional dendritic cell; CTV, cell trace violet; gMFI, geometric mean fluorescence intensity; RTOC, reaggregated thymic organ cultures; sp‑DC, splenic  conventional dendritic cell; t‑DC, thymic conventional dendritic cell; Treg cell, regulatory T cell

To mimic the impact of the thymic microenvironment on thymus‐homing peripheral cDCs, we set up RTOCs and introduced CD45^+^Lin^−^CD11c^hi^ sp‐DCs ex vivo isolated from CD45.1 congenic mice (Figure S2A–C). RTOCs with corresponding t‐DCs were set up as controls. To assess the impact of the introduced sp‐DCs on the Treg cell induction within the RTOC, we additionally introduced CTV‐labeled CD4SP Foxp3^hCD2^^−^ thymocytes isolated from adult Foxp3 reporter mice (Figure S2A,D). In this setting, the RTOCs were harvested and analyzed by flow cytometry after 4 days of culture to provide sufficient time for an efficient Treg cell induction (Figure S3). The exogenously added CD45.1^+^ cDCs could be easily distinguished from their endogenous CD45.2^+^ counterparts and made up the majority of the Lin^−^CD11c^hi^ cDC population with a significantly higher frequency when compared to CD45.2^+^ cDCs (Figures 3A and S3A). Likewise, the exogenously added CD4SP thymocytes could be accurately distinguished from endogenous CD4SP thymocytes with the help of the CTV label and made up a slightly, yet significantly higher fraction of the entire CD4SP thymocyte population (Figures 3B and S3B). The flow cytometric analysis revealed that exogenously added t‐DCs did not have an impact on the Treg cell‐inducing capacity of the RTOCs as similar frequencies of CD25^+^Foxp3^hCD2+^ Treg cells were observed among both the endogenous (CTV^−^) and exogenously added (CTV^+^) CD4SP thymocytes when compared to the RTOCs not receiving additional cDCs (Figure 3C,D, left). Importantly, the addition of sp‐DCs, which are known to possess a poor Treg cell induction capacity when compared to t‐DCs,^16^ did not result in a reduction of the Treg cell frequency in the RTOCs as comparable frequencies of CD25^+^Foxp3^hCD2+^ Treg cells were observed among both endogenous and exogenously added CD4SP thymocytes (Figure 3C,D, left). In the same line, similar frequencies of CD25^−^Foxp3^hCD2+^ Treg cell precursors (Foxp3^+^ TregP) were found among both endogenous as well as exogenously added CD4SP thymocytes in RTOCs that either only received CD4SP thymocytes or additionally also t‐DCs or sp‐DCs (Figure 3C,D, middle). In contrast, endogenous CD25^+^Foxp3^hCD2^^−^ Treg cell precursor (CD25^+^ TregP) were slightly but significantly reduced in RTOCs containing additional t‐DCs, while frequencies of CD25^+^ TregP among exogenously added CD4SP thymocytes were mildly yet significantly decreased in RTOCs containing additional sp‐DCs (Figure 3C,D, right). Furthermore, analysis of the proliferation of the exogenously added CD4SP thymocytes revealed that the addition of t‐DCs or sp‐DCs to the RTOC had no impact on the proliferation of neither newly generated CD25^+^Foxp3^hCD2+^ Treg cells nor the two Treg cell precursor populations as indicated by comparable geometric mean fluorescence intensities of the CTV label (Figure 3E).

Taken together, our data indicate that, although the introduced sp‐DCs constitute the majority of the total CD11c^hi^ cDC population within the RTOCs, they did not promote the intrathymic Treg cell induction. This finding suggests that the thymic microenvironment can gradually modulate the phenotype and functional properties of thymus‐homing peripheral cDCs.

### 3.3 The thymic microenvironment modulates the transcriptome of sp‐DCs

**Figure 4:**
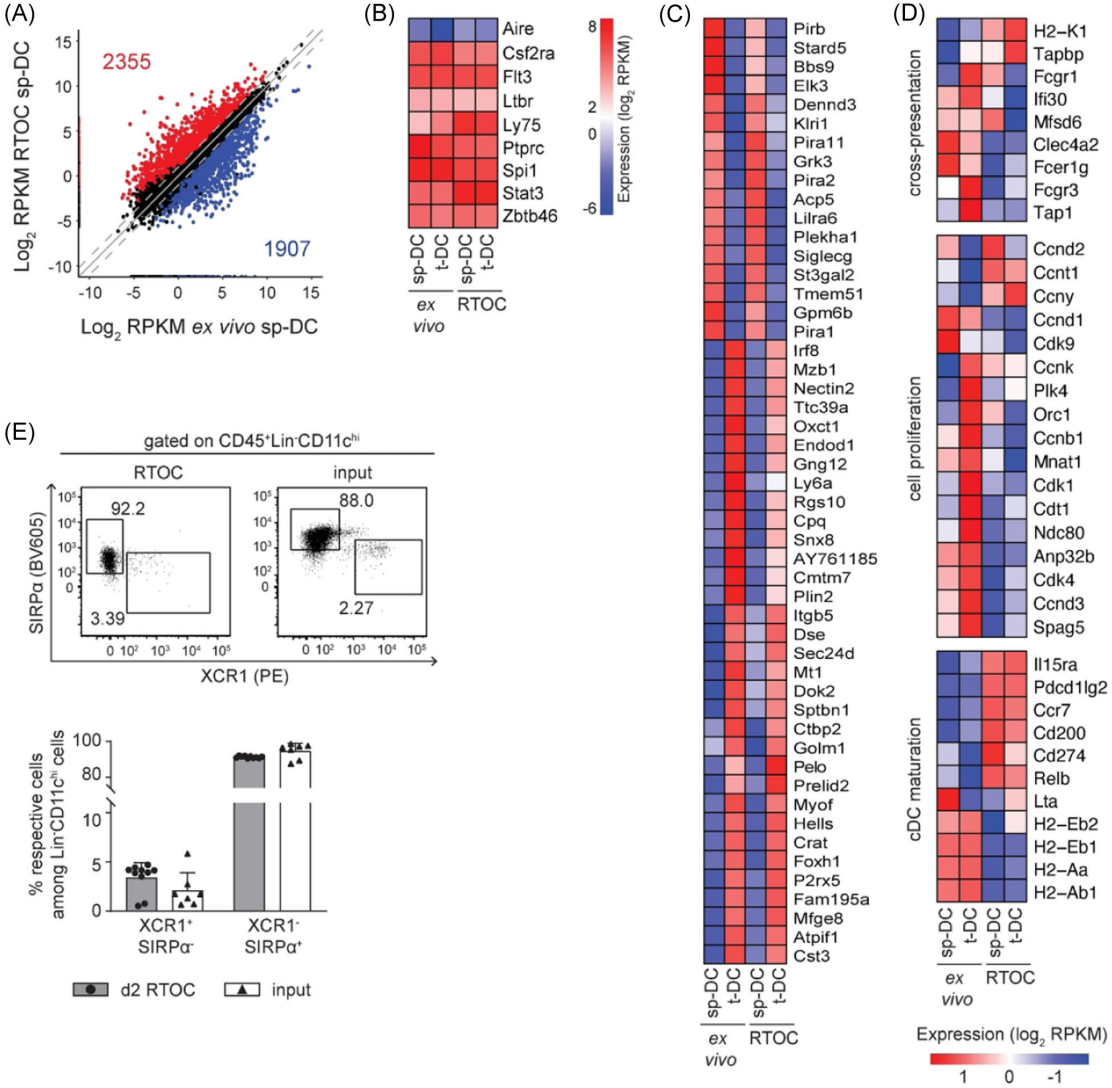
Differences between sp‐DCs isolated ex vivo or reisolated from RTOCs are observed on a transcriptomic level. RTOCs were generated from single‐cell suspensions of pooled thymi isolated from E14.5 to E16.5 fetuses of Foxp3^hCD2^ reporter mice (BALB/c background), and sp‐DCs or t‐DCs sorted from 4‐ to 8‐week‐old female CD45.1xBALB/c mice were introduced. After 2 days, 1–2 × 10^3^ cDCs were reisolated from RTOCs as CD45.1^+^Lin^−^CD11c^hi^ cells by FACS, and total RNA for transcriptional profiling by low‐input RNAseq was isolated. Total RNA from cDCs isolated ex vivo from 4‐ to 8‐week‐old female CD45.1xBALB/c mice was used as control. (A) Scatter plot highlighting the DEGs between ex vivo sp‐DCs and RTOC sp‐DCs. The DEGs were filtered with a conservative absolute log_2_ fold change cutoff of at least 1.0 and a *p*‐value cutoff, corrected for multiple testing, of at most .05. The reads per kilobase of transcript length per million mapped reads (RPKM) values were calculated based on the variance among samples. (B) Heatmap analysis depicting genes for “cross‐presentation”, “cell proliferation”, and “cDC activation” influenced by the RTOC. Gene sets were manually curated. Bars are color‐coded according to the expression value RPKM as indicated in the expression scale. Data were mean‐centered and rows were clustered using ward.D2 clustering method. (C) Heatmap analysis for cDC signature genes that are not differentially expressed between all four cell populations investigated. Bars are color‐coded according to the expression value RPKM as indicated in the expression scale. (D) Heatmap analysis for genes differentially expressed between sp‐DCs and t‐DCs, independent from ex vivo isolation or reisolation from RTOC. Bars are color‐coded according to the expression value RPKM as indicated in the expression scale. Columns are clustered by Euclidean distances. Data were mean‐centered, and rows were clustered using the ward.D2 clustering method. (B–D) All heatmap analyses were performed on log_2_ transformed data. Each column represents an average of two to three biological replicates. (E) The expression of XCR1 and SIRPα on Lin^−^CD11c^hi^ sp‐DCs, either sorted from ex vivo cells (input) or reisolated from Day 2 RTOCs, was analyzed by flow cytometry. Representative dot plots (top) and summarizing graphs (bottom) depict the frequency of XCR1^+^SIRPα^−^ cDC1s and XCR1^−^SIRPα^+^ cDC2s among Lin^−^CD11c^hi^ sp‐DCs. For the representative dot plots, numbers indicate the frequencies of cells within the depicted gates. For the summarizing graph, data are summarized from three independent experiments (mean ± SD; *n* = 2–8 biological replicates per experiment per group; *n* = 7–10 biological replicates per group in total), and data of each subset were independently tested for significance using the Mann–Whitney test. cDC, conventional dendritic cell; DEG, differentially expressed genes; FACS, fluorescence‐activated cell sorting; RNAseq, RNA sequencing; RTOC, reaggregated thymic organ cultures; sp‑DC, splenic conventional dendritic cell; t‑DC, thymic conventional dendritic cell

Next, we assessed how the thymic microenvironment is modulating sp‐DCs on a molecular level. To this end, we set up RTOCs and introduced congenically marked ex vivo isolated CD45.1^+^Lin^−^CD11c^hi^ sp‐DCs (Figure S4A). RTOCs with introduced CD45.1^+^Lin^−^CD11c^hi^ t‐DCs served as controls. On Day 2, RTOCs were harvested and CD45.1^+^ sp‐DCs or t‐DCs were reisolated (Figure S4). In this setting, the earlier time point was chosen to study an immediate and direct impact of the thymic microenvironment on the sp‐DC phenotype. Low‐input RNAseq was performed with these reisolated cells. As additional controls ex vivo isolated CD45^+^Lin^−^CD11c^hi^ sp‐DCs and t‐DCs were transcriptionally profiled. Comparing sp‐DCs reisolated from RTOCs with ex vivo isolated sp‐DCs revealed a large number (4262) of DEGs (Figure 4A), suggesting that the thymic microenvironment has a noticeable impact on the transcriptome of sp‐DCs. Yet, we cannot exclude that the experimental design itself or the rather low sample number also impacted the differential gene expression. Indeed, a distinct fraction of these DEGs, namely 995 genes, were merely impacted by the RTOC itself (“RTOC effect”, Figure S5). In contrast, genes assigned as cDC signature genes^28^, ^29^, ^30^ were not affected by the RTOC microenvironment and equally expressed in all examined groups (Figure 4B), while a small number of genes maintained to be differentially expressed between sp‐DCs and t‐DCs even under RTOC conditions (Figure 4C). Among the genes primarily affected in their expression by the “RTOC effect”, we found for examples genes involved in cross‐presentation (e.g., *Clec4a2*, *Fcer1g*, and *Fcgr3*), cell proliferation (e.g., *Ccnt1*, *Ccny*, *Cdk4*, and *Spag5*), or DC maturation (e.g., *Cd200*, *Cd274*, and *Relb*) (Figure 4D). Interestingly, while genes related to cross‐presentation and cell proliferation were mainly negatively impacted by the RTOC, the expression of genes involved in DC maturation, with the exception of MHCII genes, were mainly increased. Importantly, the subset composition of sp‐DCs was found to be unaltered within the RTOC compared to input cells when analyzed by flow cytometry (Figures 4E and S6). Thus, although obvious limitations of the culture system exist, the RTOC studies also suggest that the commitment to the cDC1 or cDC2 lineage is a fixed characteristic, probably established already at the pre‐DC stage.^12^, ^31^

**Figure 5:**
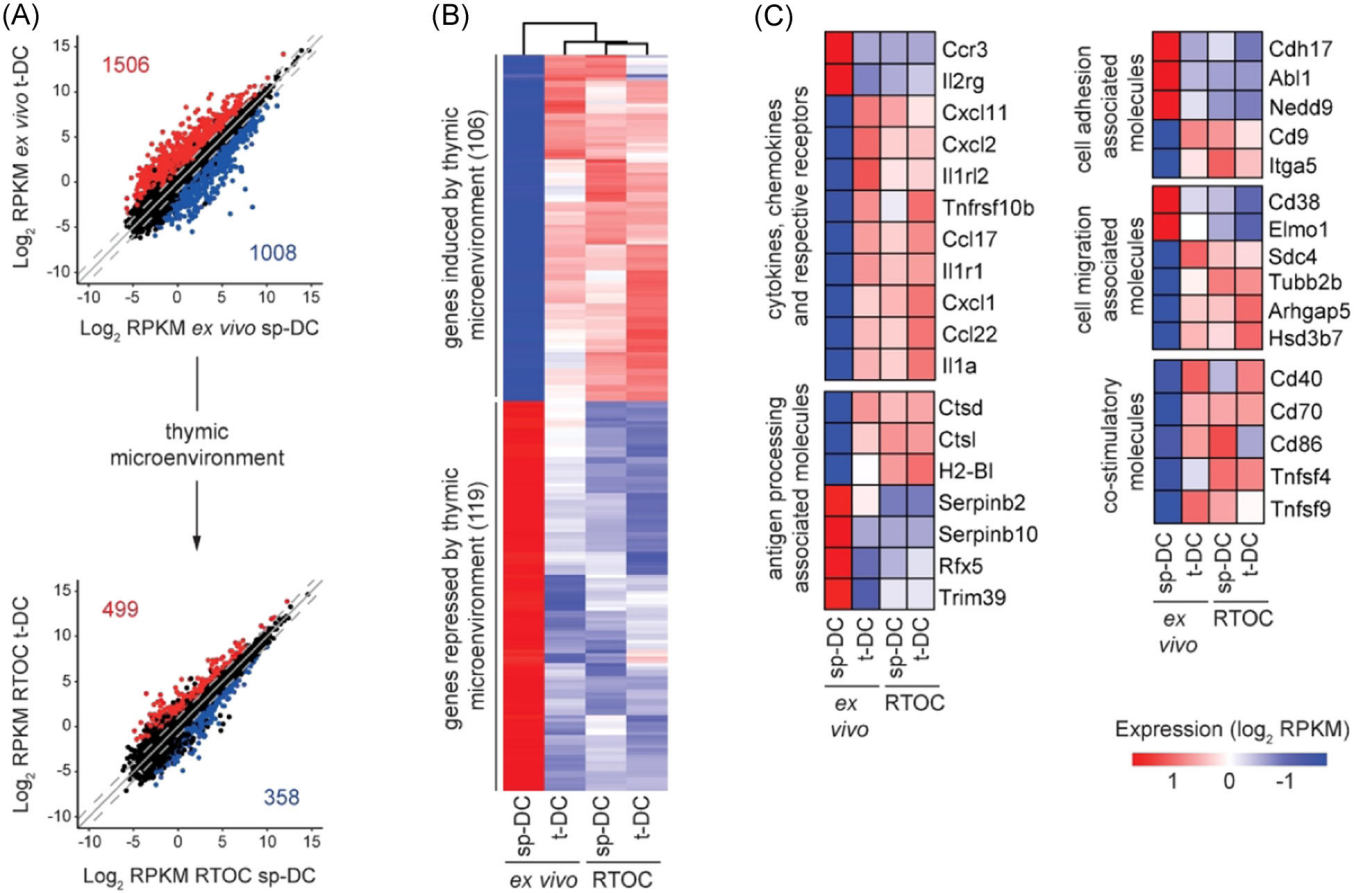
The thymic microenvironment of RTOCs alters the transcriptome of sp‐DCs. RTOCs were generated from single‐cell suspensions of pooled thymi isolated from E14.5 to E16.5 fetuses of Foxp3^hCD2^ reporter mice (BALB/c background), and sp‐DCs or t‐DCs sorted from 4‐ to 8‐week‐old female CD45.1xBALB/c mice were introduced. After 2 days, 1–2 × 10^3^ cDCs were reisolated from RTOCs as CD45.1^+^Lin^−^CD11c^hi^ cells by FACS, and total RNA for transcriptional profiling by low‐input RNAseq was isolated. Total RNA from cDCs isolated ex vivo from 4‐ to 8‐week‐old female CD45.1xBALB/c mice was used as control. (A) Scatter plots highlighting the DEGs between ex vivo cDCs and RTOC cDCs, respectively. The DEGs were filtered with a conservative absolute log_2_ fold change cutoff of at least 1.0 and a *p*‐value cutoff, corrected for multiple testing, of at most .05. The reads per kilobase of transcript length per million mapped reads (RPKM) values were calculated based on the variance among samples. (B) A total of 225 genes are shaped by the thymic microenvironment. Heatmap analysis for genes induced or repressed by the thymic microenvironment. Columns are clustered by Euclidean distances. (C) Heatmap analysis depicting genes for “cytokines, chemokines, and their respective receptors”, “antigen processing associated molecules”, “cell adhesion associated molecules”, “cell migration associated molecules”, and “co‐stimulatory molecules” influenced by the thymic microenvironment. Gene sets were manually curated. Data were mean‐centered and rows were clustered using ward.D2 clustering method. (B and C) Heatmaps are color‐coded according to the expression value RPKM as indicated in the expression scale. All heatmap analyses were performed on log_2_ transformed data. Each column represents an average of two to three biological replicates. cDC, conventional dendritic cell; DEG, differentially expressed genes; FACS, fluorescence‐activated cell sorting; RNAseq, RNA sequencing; RTOC, reaggregated thymic organ cultures; sp‑DC, splenic conventional dendritic cell; t‑DC, thymic conventional dendritic cell

In line with our previously published finding,^16^ we also observed a large number of 2514 DEGs when comparing ex vivo isolated sp‐DCs and t‐DCs (Figure 5A). Interestingly, only 857 DEGs were observed between sp‐DCs and t‐DCs reisolated from RTOCs, implicating that the thymic microenvironment in RTOCs has a noticeable impact on the transcriptome of sp‐DCs, markedly reduced the transcriptional differences between t‐DCs and sp‐DCs, and finally, conferred a transcriptomic profile that more closely resembled the one from t‐DCs. To identify the genes that were selectively modulated in sp‐DCs by the thymic microenvironment of the RTOC, we first determined the overlap of the “modulated sp‐DC signature” (i.e., genes likewise up‐ or downregulated within sp‐DCs reisolated from RTOCs and ex vivo isolated t‐DCs when compared to ex vivo isolated sp‐DCs) with the so‐called “t‐DC core signature”, genes that were not differentially regulated between ex vivo isolated t‐DCs and t‐DCs reisolated from RTOCs (Figure S7A). Subsequently, from this overlap, all genes that were merely modulated by the RTOC condition itself, the so‐called “RTOC effect”, were eliminated. This “RTOC effect” was defined as the overlap between three groups of genes: (1) genes commonly expressed in sp‐DCs and t‐DCs reisolated from RTOCs, (2) genes differentially regulated between ex vivo isolated t‐DCs and t‐DCs reisolated from RTOC, and (3) genes differentially regulated between ex vivo isolated sp‐DCs and sp‐DCs reisolated from RTOC (Figure S5). By this strict filtering process, 225 genes were identified that were either induced (106) or repressed (119) in sp‐DCs by the thymic microenvironment (Figures 5B and S7B). Many of these genes encode immunologically relevant molecules, including “cytokines, chemokines, and their respective receptors” (e.g., *Ccl17*, *Ccl22*, *Il1r1*, *Il1a*), “antigen processing associated molecules” (e.g., *Ctsd*, *Ctsl*, *Serpinb2*, and *Serpinb10*), “cell adhesion associated molecules” (e.g., *Nedd9*, *Cd9*, and *Itga5*), “cell migration associated molecules” (e.g., *Cd38*, *Elmo1*, and *Tubb2b*), and “costimulatory molecules” (e.g., *Tnfsf9*, *Tnfsf4*, *Cd40*, *Cd70*, and *Cd86*) (Figure 5C). Together, these results indicate that the thymic microenvironment can gradually modulate thymus‐homing peripheral cDCs on the molecular level by driving the transcriptomic profile of sp‐DCs towards the one of t‐DCs, thereby transferring thymus‐specific properties to the newly entering cDCs from the periphery.

## 4 DISCUSSION

Thymic cDCs play an essential role in key processes of central tolerance like the clonal deletion of self‐reactive thymocytes and the generation of Treg cells.^2^, ^4^ This important contribution to central tolerance was demonstrated for both thymus‐resident CD8α^+^SIRPα^−^ t‐DCs, which develop intrathymically,^8^, ^9^ as well as for migratory CD8α^−^SIRPα^+^ t‐DCs, which home to the thymus from the periphery via CCR2‐mediated chemotaxis and α_4_ integrin‐dependent adhesion.^13^, ^18^, ^32^ Accordingly, we have previously demonstrated that bulk t‐DCs possess a superior Treg cell‐inducing capacity when compared to sp‐DCs, leading to the differentiation of stable Foxp3^+^ Treg cells.^16^ This raised the question of whether sp‐DCs, which already have undergone a number of differentiation steps,^33^, ^34^ are still plastic and can be modulated by the local microenvironment after entry into the thymus to acquire the unique functional properties of t‐DCs, including the superior Treg cell‐inducing capacity. This point is particularly relevant as several studies have reported diverging transcriptional profiles of cDCs from different lymphoid organs,^35^, ^36^ suggesting that milieu‐specific programs manifest in these different cDCs.

In the present study, we have further addressed this important question experimentally by employing RTOCs to accurately introduce sp‐DCs into the thymic microenvironment. For this purpose, RTOCs were carefully characterized regarding their cellular composition, confirming that they mature over time and successfully support thymocyte and TEC development. Importantly, we further only used ex vivo isolated cDCs from unmanipulated mice, while previously published studies have only investigated the thymus‐homing and subsequent maturation of circulating cDCs by adoptively transferring sp‐DCs derived from donor mice that were exposed to Flt3L‐secreting B16 melanoma cells to expand the cDC population.^17^, ^32^ Our results suggest that sp‐DCs can acquire a thymus‐specific functionality, as the addition of an unmanipulated sp‐DC population with a physiological subset composition does not negatively impact the Treg cell frequency, although sp‐DCs were shown to commonly possess only a low Treg cell‐inducing capacity and although these introduced cells constituted the major proportion of total cDCs within the RTOC. These indirect findings suggest that sp‐DCs can be modulated by the thymic microenvironment and acquire an improved Treg cell‐inducing capacity. Otherwise, the Treg cell frequencies within RTOCs containing the added sp‐DCs should have been severely decreasing due to the generally poor Treg cell‐inducing capacity of sp‐DCs.^16^ Interestingly, our results from the control RTOCs, which harbored exogenous t‐DCs, further showed that the introduction of additional t‐DCs does not increase the Treg cell frequency. This finding is in line with studies demonstrating that the thymic microenvironment creates a saturable niche for the Treg cell development, which is tightly controlled in a TCR‐instructive manner by the availability of interleukin‐2.^37^, ^38^, ^39^ However, as neither the endogenous cDCs nor any other population of the thymic hematopoietic stromal cells (THSCs), such as macrophages or plasmacytoid DCs, was depleted from the RTOC, an impact of those cells on the Treg cell frequency and, thus, a redundant role of the added cDCs cannot be formally excluded. Yet, a considerable impact of any of these populations seems unlikely, because cDCs were shown to constitute the most efficient THSC population with regard to supporting Treg cell development, and exogenously added cDCs made up the vast majority of total cDCs within RTOCs.^40^ Moreover, mTECs, which are known to possess a considerable Treg cell‐inducing capacity, were found to exhibit a delayed development within RTOCs and constitute only a minor population in these reaggregated cultures.^41^, ^42^ Thus, mTECs very likely are not of major relevance for the Treg cell differentiation within the investigated RTOC system.

The molecular profiling of sp‐DCs reisolated from RTOCs implied that the thymic microenvironment tuned the transcriptome of sp‐DCs towards the transcriptome of t‐DCs already after 2 days of culture. Yet, it cannot be exclude that the experimental design itself or the rather low sample number may also impacted the differential gene expression. Of note, cDC signature genes and other genes that are relevant for general biological and cellular processes were not changed by the thymic microenvironment. In addition, our findings support the notion that the subset composition of cDCs is not influenced by the thymus‐specific milieu, and commitment to the cDC1 or cDC2 lineage probably occurs already at the pre‐DC stage.^12^, ^31^ Among the genes, which were modulated in sp‐DCs by the thymic microenvironment, *Ccl17* and *Ccl22* might be of particular relevance as Proietto et al.^43^ showed that these two chemokines were expressed at very high levels only by CD8α^−^SIRPα^+^ t‐DCs, and the supernatant of CD8α^−^SIRPα^+^ t‐DCs efficiently attracted CD4SP thymocytes in trans‐well migration assays. Importantly, CCR4, the receptor for CCL17 and CCL22, is expressed on postpositive selection DP and immature CD4SP thymocytes in the medulla and is known to be required for interactions between medullary cDCs and thymocytes.^44^ Hence, the rapid and strong upregulation of *Ccl17* and *Ccl22* expression on sp‐DCs within the thymic microenvironment might confer a higher potency for the clonal deletion of self‐reactive thymocytes and the generation of Treg cells through these modulated sp‐DCs.

In addition, several costimulatory molecules like *Cd40*, *Cd70*, *Tnfsf9*, *Tnfsf4*, and *Cd86*, which are known to be essential for the development of Treg cells,^14^, ^45^, ^46^, ^47^ were upregulated on sp‐DC reisolated from RTOCs. Upregulation of these costimulatory molecules follows the steady‐state maturation process of t‐DCs, which is induced in CD8α^−^SIRPα^+^ migratory t‐DCs only upon thymic entry.^11^, ^17^ Importantly, only matured cDCs possess the capacity to contribute to key processes of central tolerance.^48^ For the intrathymic maturation and homeostasis of cDCs, mTECs were reported to play an only minor role.^49^, ^50^ Thus, although it is known that the heterogeneity of TECs is completed only in adulthood,^51^, ^52^ it is unlikely that the observed clear differences in the TEC compartment between embryonic thymi in RTOCs and ex vivo isolated adult thymi have a considerable effect on the modulation of sp‐DCs. By contrast, different groups recently proposed that cognate interactions of antigen‐specific CD4SP thymocytes with both resident and migratory t‐DCs efficiently support the homeostatic maturation of t‐DCs.^17^, ^50^ However, in our study, the frequency of CD4SP thymocytes was still rather low in RTOCs harvested on Day 2, even though frequencies rapidly increased and reached levels comparable to those observed in ex vivo isolated adult thymi by Day 4. Thus, it is likely that the initial maturation and adaptation processes are seen in sp‐DCs reisolated from RTOCs at Day 2 might be imparted by additional mechanisms, while the mechanism involving CD4SP thymocytes might account for the progressed maturation of sp‐DCs and acquisition of thymus‐specific functional properties after 4 days of culture. Accordingly, Oh et al.^17^ and Spidale et al.^50^ did not rule out the contribution of CD8SP and DP thymocytes to the intrathymic maturation of cDCs. As these cells are already present in RTOCs at Day 2 with a frequency comparable to that found in ex vivo isolated adult thymi, they might play a role in the abovementioned initial maturation and adaptation processes. This scenario seems especially likely for DP thymocytes, as they express the chemokine receptor CCR4, like CD4SP thymocytes, and are, thus, attracted by CCL17 and CCL22,^44^ which we found to be highly induced in sp‐DCs reisolated from RTOCs.

Viewed as a whole, the data from the present study indicate that the thymic microenvironment can moderately modulate the phenotype of sp‐DCs, which is likely accompanied by an adjusted Treg cell‐inducing capacity. Thus, besides their already advanced differentiation stage cDCs from secondary lymphoid organs actually retain remarkable plasticity, which allows for modulation by the thymic microenvironment. Future studies are required to unravel the exact molecular mechanisms and the precise timing of the intrathymic modulation of thymus‐homing peripheral cDCs.

## CONFLICT OF INTERESTS

The authors declare that there are no conflict of interests.

## AUTHOR CONTRIBUTIONS

Susanne Herppich performed the experiments. Susanne Herppich and Michael Beckstette analyzed the data. Susanne Herppich and Jochen Huehn designed the research, interpreted the data, and wrote the manuscript.

## Supporting information

Supporting information.

## Data Availability

RNA sequencing data can be accessed at NCBI Gene Expression Omnibus under the accession number GSE164280 (www.ncbi.nlm.nih.gov/geo/query/acc.cgi?acc=GSE164280).
